# The Cat and Mouse of HIV-1 Antibody Escape

**DOI:** 10.1371/journal.ppat.1000592

**Published:** 2009-09-18

**Authors:** John R. Mascola

**Affiliations:** Vaccine Research Center, National Institute of Allergy and Infectious Diseases, National Institutes of Health, Bethesda, Maryland, United States of America; University of Notre Dame, United States of America

Human immunodeficiency virus type 1 (HIV-1) is a chronically replicating lentivirus that must escape from adaptive immune responses that arise during the course of infection. Viral persistence is maintained by the rapid rate of HIV-1 replication and the error-prone reverse transcription of the viral genome, which produces viral variants that continually escape antibody and cytotoxic T cell responses [1]–[3]. Antibodies directed against the gp120 and gp41 components of the viral envelope glycoprotein (Env) develop within the first few weeks of infection [4],[5], but antibodies that can neutralize the infecting virus (NAbs) are usually not detected until more than 12 weeks after HIV-1 infection [6]. Thus, in natural HIV-1 infection, NAbs are not believed to play a major role in containing the acute phase of HIV-1 replication. However, several studies have shown that once NAbs arise, they exert immune selection pressure on the viral quasispecies [7]–[14].

Viral escape from autologous NAbs was first described in lentiviral infections of several animal species [15]–[17]. For example, the successive waves of viremia in horses caused by equine infectious anemia virus are thought to be due to the sequential development of viral variants that temporarily evade the host NAb response. HIV-1 escape from autologous NAbs was first described in the early 1990s [18]–[20]. Subsequently, numerous research groups showed that plasma antibodies from a time point contemporaneous with viral isolation did not neutralize the autologous virus, and that NAbs against the isolated virus developed only months later [7]–[14],[21],[22]. Thus, the NAb response continually lags behind viral replication. The initial studies of NAb escape were limited by the inefficiency of isolating replication competent HIV-1 from patient plasma or lymphocytes. The more recently performed studies used molecularly cloned Env-pseudoviruses to more robustly study the plasma viral quasispecies at sequential time points. These data confirmed that, at any given time point during the course of HIV-1 infection, the circulating quasispecies of viral variants is resistant to the circulating plasma NAb. At first glance, these findings might suggest that HIV-1 should become progressively more resistant to neutralization over time. Interestingly, this is not the case. HIV-1 isolates that are resistant to circulating autologous NAbs generally remain sensitive to neutralization by several known monoclonal antibodies (mAbs) or by heterologous plasma obtained for other individuals with HIV-1. This has led to several key questions related to autologous virus NAb escape: What are the Env epitopes targeted by early autologous NAbs and how does the virus escape from these NAbs? How does continuous neutralization escape occur without leading to global changes in viral neutralization sensitivity? Finally, what are the implications of NAb escape for HIV-1 vaccines?

In this issue of *PLoS Pathogens*, two teams of investigators provide some initial answers to these questions [23],[24]. Both groups utilized clinical samples collected from seroconversion cohorts of individuals with subtype C HIV-1. The investigators studied the development of the autologous NAb response from the acute phase, though the first 2 years of infection. A limiting dilution PCR methodology was used to clone and study HIV-1 variants from sequential plasma samples over time. Moore and colleagues studied four individuals and found that the early NAb response was restricted to two epitopes on the HIV-1 Env. They used chimeric viral clones and site-specific mutagenesis to define an epitope composed of the first and second variable region (V12) of the HIV-1 Env. A second epitope was identified within a variable alpha-2 helix region of Env that is just past the V3 loop. The restricted nature of the autologous NAb response to variable Env regions is an important finding, because it helps to explain how the virus can readily mutate to evade the NAb response. The V12 region in particular can tolerate insertions and deletions of amino acid residues without sacrificing Env function. In addition, specific amino acid changes and alterations in glycosylation in these two epitopes were found to be associated with neutralization escape. In one individual, the development of a NAb response to the alpha-2 helix region was associated with a 7-fold drop in plasma viremia, and a 4-fold rebound as neutralization escape occurred. Rong and colleagues similarly studied longitudinal samples from two individuals and found a highly restricted set of NAbs. They also identified the V12 region as a key target of autologous NAbs. Mapping studies demonstrated that specific amino acid sequence alterations, as well as changes in the pattern of glycosylation, were important components of neutralization escape. Importantly, they were able to isolate two mAbs from one patient, and demonstrated that a single amino acid substitution affecting a glycosylation site in V2 was responsible for resistance to these mAbs. In some cases, mutations outside of the specific neutralization epitopes were also associated with neutralizing escape. Given the complex trimeric structure of the HIV-1 Env, it is well known that distant mutations can affect the conformational structure of Env and impact antibody recognition of an epitope [25]. While these two new studies have probably not described the full spectrum of autologous NAb responses, the consistent finding of an early dominant NAb response to one or two variable regions of Env that can vary without major cost to viral fitness does help explain how the virus is able to effectively evade the NAb response.

The study of the early autologous NAb response adds to our understanding of the role of NAbs in natural HIV-1 infection, and has potential implications for HIV-1 vaccine design. We know that, over time, more broadly reactive NAbs develop in some individuals with HIV-1 [26]–[28]. These NAbs appear to target functionally conserved regions of Env such as the receptor or co-recpetor binding sites, or conserved regions of gp41 [27], [29]–[31]. Thus, immune escape from such NAbs would, in theory, be much more difficult [32]. In addition, these antibodies can protect against AIDS virus infection in non-human primate models [33],[34]. We still do not understand why such NAbs arise so late during the course of HIV-1 infection. Hence, investigators should continue to study the longer-term evolution of the NAb response in order to better understand the early epitope dominance of the autologous NAb response, and the clinical and virologic factors associated with the evolution from a type-restricted NAb response to a more broadly reactive response. While NAbs may arise too late during natural HIV-1 infection to have a major impact on HIV-1 replication, a major goal of vaccine researchers is to generate pre-existing NAb responses that can prevent initial HIV-1 infection, or contain the virus during the initial phase of viral dissemination [3],[26],[35],[36]. A better understanding of the evolution of the natural NAb response during natural infection, including the viral epitopes targeted, can provide insights for vaccine immunogen design.

## References

[1] Pantaleo G, Koup RA (2004). Correlates of immune protection in HIV-1 infection: what we know, what we don't know, what we should know.. Nat Med.

[2] Letvin NL (2006). Progress and obstacles in the development of an AIDS vaccine.. Nat Rev Immunol.

[3] Montefiori D, Sattentau Q, Flores J, Esparza J, Mascola J (2007). Antibody-based HIV-1 vaccines: recent developments and future directions.. PLoS Med.

[4] Moore JP, Cao Y, Ho DD, Koup RA (1994). Development of the anti-gp120 antibody response during seroconversion to human immunodeficiency virus type 1.. J Virol.

[5] Tomaras GD, Yates NL, Liu P, Qin L, Fouda GG (2008). Initial B-cell responses to transmitted human immunodeficiency virus type 1: virion-binding immunoglobulin M (IgM) and IgG antibodies followed by plasma anti-gp41 antibodies with ineffective control of initial viremia.. J Virol.

[6] Montefiori DC, Morris L, Ferrari G, Mascola JR (2007). Neutralizing and other antiviral antibodies in HIV-1 infection and vaccination.. Curr Opin HIV AIDS.

[7] Richman DD, Wrin T, Little SJ, Petropoulos CJ (2003). Rapid evolution of the neutralizing antibody response to HIV type 1 infection.. Proc Natl Acad Sci U S A.

[8] Wei X, Decker JM, Wang S, Hui H, Kappes JC (2003). Antibody neutralization and escape by HIV-1.. Nature.

[9] Gray ES, Moore PL, Choge IA, Decker JM, Bibollet-Ruche F (2007). Neutralizing antibody responses in acute human immunodeficiency virus type 1 subtype C infection.. J Virol.

[10] Mahalanabis M, Jayaraman P, Miura T, Pereyra F, Chester EM (2009). Continuous viral escape and selection by autologous neutralizing antibodies in drug-naive human immunodeficiency virus controllers.. J Virol.

[11] Frost SD, Wrin T, Smith DM, Kosakovsky Pond SL, Liu Y (2005). Neutralizing antibody responses drive the evolution of human immunodeficiency virus type 1 envelope during recent HIV infection.. Proc Natl Acad Sci U S A.

[12] Bunnik EM, Pisas L, van Nuenen AC, Schuitemaker H (2008). Autologous neutralizing humoral immunity and evolution of the viral envelope in the course of subtype B human immunodeficiency virus type 1 infection.. J Virol.

[13] Moore PL, Gray ES, Choge IA, Ranchobe N, Mlisana K (2008). The c3-v4 region is a major target of autologous neutralizing antibodies in human immunodeficiency virus type 1 subtype C infection.. J Virol.

[14] Li B, Decker JM, Johnson RW, Bibollet-Ruche F, Wei X (2006). Evidence for potent autologous neutralizing antibody titers and compact envelopes in early infection with subtype C human immunodeficiency virus type 1.. J Virol.

[15] Cheevers WP, Knowles DP, Norton LK (1991). Neutralization-resistant antigenic variants of caprine arthritis-encephalitis lentivirus associated with progressive arthritis.. J Infect Dis.

[16] Salinovich O, Payne SL, Montelaro RC, Hussain KA, Issel CJ (1986). Rapid emergence of novel antigenic and genetic variants of equine infectious anemia virus during persistent infection.. J Virol.

[17] Montelaro RC, Parekh B, Orrego A, Issel CJ (1984). Antigenic variation during persistent infection by equine infectious anemia virus, a retrovirus.. J Biol Chem.

[18] Arendrup M, Nielsen C, Hansen JE, Pedersen C, Mathiesen L (1992). Autologous HIV-1 neutralizing antibodies: emergence of neutralization-resistant escape virus and subsequent development of escape virus neutralizing antibodies.. J Acquir Immune Defic Syndr.

[19] Albert J, Abrahamsson B, Nagy K, Aurelius E, Gaines H (1990). Rapid development of isolate-specific neutralizing antibodies after primary HIV-1 infection and consequent emergence of virus variants which resist neutralization by autologous sera.. Aids.

[20] Montefiori DC, Zhou IY, Barnes B, Lake D, Hersh EM (1991). Homotypic antibody responses to fresh clinical isolates of human immunodeficiency virus.. Virology.

[21] Bradney AP, Scheer S, Crawford JM, Buchbinder SP, Montefiori DC (1999). Neutralization escape in human immunodeficiency virus type 1-infected long-term nonprogressors.. J Infect Dis.

[22] Deeks SG, Schweighardt B, Wrin T, Galovich J, Hoh R (2006). Neutralizing antibody responses against autologous and heterologous viruses in acute versus chronic human immunodeficiency virus (HIV) infection: evidence for a constraint on the ability of HIV to completely evade neutralizing antibody responses.. J Virol.

[23] Rong R, Li B, Lynch RB, Haaland RE, Murphy MK (2009). Escape from autologous neutralizing antibodies in acute/early subtype C HIV-1 infection requires multiple pathways.. PLoS Pathog.

[24] Moore PL, Ranchobe N, Lambson BE, Gray ES, Cave E (2009). Limited neutralization antibody specificities drive neutralization escape in early HIV-1 subtype C infection.. PLoS Pathog.

[25] Pantophlet R, Burton DR (2006). GP120: target for neutralizing HIV-1 antibodies.. Annu Rev Immunol.

[26] Stamatatos L, Morris L, Burton DR, Mascola JR (2009). Neutralizing antibodies generated during natural HIV-1 infection: good news for an HIV-1 vaccine?. Nat Med.

[27] Sather DN, Armann J, Ching LK, Mavrantoni A, Sellhorn G (2009). Factors associated with the development of cross-reactive neutralizing antibodies during human immunodeficiency virus type 1 infection.. J Virol.

[28] Doria-Rose NA, Klein RM, Manion MM, O'Dell S, Phogat A (2009). Frequency and phenotype of human immunodeficiency virus envelope-specific B cells from patients with broadly cross-neutralizing antibodies.. J Virol.

[29] Binley JM, Lybarger EA, Crooks ET, Seaman MS, Gray E (2008). Profiling the specificity of neutralizing antibodies in a large panel of plasmas from patients chronically infected with human immunodeficiency virus type 1 subtypes B and C.. J Virol.

[30] Li Y, Migueles SA, Welcher B, Svehla K, Phogat A (2007). Broad HIV-1 neutralization mediated by CD4-binding site antibodies.. Nat Med.

[31] Li Y, Svehla K, Louder MK, Wycuff D, Phogat S (2009). Analysis of neutralization specificities in polyclonal sera derived from human immunodeficiency virus type 1-infected individuals.. J Virol.

[32] Trkola A, Kuster H, Rusert P, von Wyl V, Leemann C (2008). In vivo efficacy of human immunodeficiency virus neutralizing antibodies: estimates for protective titers.. J Virol.

[33] Mascola JR (2003). Defining the protective antibody response for HIV-1.. Curr Mol Med.

[34] Burke B, Barnett SW (2007). Broadening our view of protective antibody responses against HIV.. Curr HIV Res.

[35] Burton DR, Desrosiers RC, Doms RW, Koff WC, Kwong PD (2004). HIV vaccine design and the neutralizing antibody problem.. Nat Immunol.

[36] Haynes BF, Montefiori DC (2006). Aiming to induce broadly reactive neutralizing antibody responses with HIV-1 vaccine candidates.. Expert Rev Vaccines.

